# Dissecting of the AI-2/LuxS Mediated Growth Characteristics and Bacteriostatic Ability of *Lactiplantibacillus plantarum* SS-128 by Integration of Transcriptomics and Metabolomics

**DOI:** 10.3390/foods11050638

**Published:** 2022-02-22

**Authors:** Yilin Qian, Yuan Li, Tengteng Xu, Huijuan Zhao, Mingyong Zeng, Zunying Liu

**Affiliations:** College of Food Science and Engineering, Ocean University of China, Qingdao 266003, China; qianyl92@outlook.com (Y.Q.); liyuan@ouc.edu.cn (Y.L.); xtteng924@163.com (T.X.); zhj13583036006@163.com (H.Z.); mingyz@ouc.edu.cn (M.Z.)

**Keywords:** quorum sensing, *Lactiplantibacillus plantarum*, bacteriostatic ability, transcriptomics, metabolomics

## Abstract

*Lactiplantibacillus plantarum* could regulate certain physiological functions through the AI-2/LuxS-mediated quorum sensing (QS) system. To explore the regulation mechanism on the growth characteristics and bacteriostatic ability of *L. plantarum* SS-128, a *luxS* mutant was constructed by a two-step homologous recombination. Compared with Δ*luxS*/SS-128, the metabolites of SS-128 had stronger bacteriostatic ability. The combined analysis of transcriptomics and metabolomics data showed that SS-128 exhibited higher pyruvate metabolic efficiency and energy input, followed by higher LDH level and metabolite overflow compared to Δ*luxS*/SS-128, resulting in stronger bacteriostatic ability. The absence of *luxS* induces a regulatory pathway that burdens the cysteine cycle by quantitatively drawing off central metabolic intermediaries. To accommodate this mutations, Δ*luxS*/SS-128 exhibited lower metabolite overflow and abnormal proliferation. These results demonstrate that the growth characteristic and metabolism of *L. plantarum* SS-128 are mediated by the AI-2/LuxS QS system, which is a positive regulator involved in food safety. It would be helpful to investigate more bio-preservation control potential of *L. plantarum*, especially when applied in food industrial biotechnology.

## 1. Introduction

Lactic acid bacteria (LAB) comprise a huge group of safe and widespread microorganisms in nature, and they are primarily applied as starter cultures and probiotics [1,2]. LAB are apprehended as ideal candidates for commercial exploitation in food industry with their status recognized as Generally Regarded As Safe (GRAS) and Qualified Presumption of Safety (QPS) in the European Union [3,4]. Aside from the health-promoting and probiotic properties, certain LAB producing organic acids, including lactic acid and phenyllactic acid (PLA), are also associated with food industrial biotechnology, providing food preservation for biocatalysis [5,6,7,8,9]. Today’s food industry faces the tremendous problem in producing goods that are not only productive, but also wholesome for customers, as well as longer-lasting [4,5]. Consequently, the scientific research in the potential of LAB as bio-preservatives has attracted continuous interest. Strategies of enhancement on bio-preservation potential can be roughly classified into two major types: the strain development strategy and microbiological control strategy. The strain development strategy involves natural approaches or metabolic engineering, and it is used for strain transformation. The microbiological control strategy involves the regulation of LAB to microorganisms throughout the food system or environment. Both strategies are regulated by quorum sensing (QS), which provides new opportunities to enhance the safety and quality of foods by the “positive regulation” of QS.

QS, associated with some cellular activities, is a process in which cells socially coordinate gene expression, involving producing extracellular signaling molecules called autoinducers [10,11,12]. The QS mediated by autoinducer-2 (AI-2)/LuxS could be detected in various Gram-negative and -positive organisms [13,14]. The gene *luxS* converts S-ribosomal homocysteine (SRH) into homocysteine and 4,5-dihydroxy-2,3-pentanedione (DPD) that undergoes spontaneous rearrangements to form AI-2. The exogenous addition of AI-2 could show influences on the biofilm formation, bacteriostatic action, and stress tolerance by LAB have been reported [14,15,16,17,18]. However, to enhance the “positive regulation” of QS in LAB, focusing on the metabolic pathway regulated by *luxS* could be an effective way compared with the addition of expensive signaling molecules. Previous studies have shown that the inactivation of the *luxS* gene has shown greatly impacts on the *Lactobacillus reuteri* 100-23C behaviors, which are inhabited in mice intestinal tracts [19]. Lebeer et al. reported that the *luxS* mutation could show indirect impact on biofilm formation in *Lactobacillus rhamnosus* GG, while it was not impacted by exogenous AI-2 mediated QS [20]. The role of the *luxS* mediated molecular mechanisms from the proteomic analyses in bacteriocin production was reported by Jia et al. [21]. However, the growth characteristic of the wild strain and the *luxS* mutant in the above studies showed inconsistent trends. Meanwhile, the regulation of AI-2/LuxS on LAB growth characteristics and bacteriostatic ability has not been elucidated yet.

*L. plantarum* possesses the ability of rapid proliferation, due to the strong resistance against the environment of complex food matrix, indicating its high potential for industrial application [16,22]. Unlike other strains, *L. plantarum* can synthesize homocysteine from cysteine by direct sulfhydrylation involving the *cysK* enzyme, to ensure the regeneration of the methyl group of S-adenosyl methionine (SAM) [20]. Since the unique functions of the *cysK* enzyme carried out are pretty similar to the *luxS*, we hypothesized that it contributes to enhancing the growth ability and stress resistance of *L. plantarum*, and also helps to focus on the *luxS*.

Previously, we found that the cooperation of *L. plantarum* showed high biopreservative activity, which was related to the AI-2/LuxS system [23], but the role of the *luxS* system has not been clearly elucidated. In this study, we aimed to understand the regulation mechanism of the AI-2/LuxS-mediated QS system on the growth characteristics and bacteriostatic ability of LAB, to provide a basis for improving its bio-preservation function.

## 2. Materials and Methods

### 2.1. Construction of luxS-Mutant of SS-128

The *luxS*-mutant of SS-128, named Δ*luxS*/SS-128, was constructed using fusion PCR and a two-step homologous recombination. In brief, the *luxS* gene-flanked two fragments were amplified and fused by overlap extension PCR with a primer pair. The primers are shown in Table 1. The ligation products, transforming into competent *Escherichia coli* pFED760, generated the recombinant plasmids, which carried the homologous fragments for allelic exchange of flanking fragments of the *luxS* gene. The pFED760 was a temperature-sensitive plasmid and was a kind gift from Professor Yiyong Luo (Kunming University of Science and Technology, Kunming, China). Recombinant plasmids pFED760-*luxS* were extracted and transformed into SS-128 by electroporation. The Δ*luxS*/SS-128 was constructed through a two-step homologous recombination by changing the temperature to control the replication and suicide of pFED760.

### 2.2. AI-2 Assay

The AI-2 bioluminescence assay was operated according to the approach with some modifications [24]. The *Vibrio harveyi* strain BB170 was cultured in AB medium at 30 °C for 12 h and diluted with the AB medium at a ratio of 1:5000. The medium was added to the diluted *V. harveyi* culture at a final concentration of 10% (*v*/*v*). The white 96-well plates vibrated at 100 rpm at 30 °C (PBS was used as the negative control). Light production was recorded every 30 min using a Synergy H4 hybrid microplate reader (Bio-Tek, Winooski, VT, USA) in the bioluminescence mode. AI-2 activity was calculated as the difference compared with the bioluminescence level in the control group and presented as relative luminescence units (RLUs).

The total RNA was directly extracted from *L. plantarum* according to the approach described by Zhang et al. at incubation times of 6, 8, 10, 12, 14, 16, 20, 24, 36, 48 h, respectively [25]. The bacteria were collected after being washed twice with PBS. According to the protocol, the obtained precipitate was used for total RNA extraction with TRIzol Reagent (Invitrogen, Carlsbad, CA, USA). The quality of the RNA was detected through the agarose gel electrophoresis, and the concentration was measured through the NanoDrop spectrophotometer (Nano-200, Hangzhou Austrian Sheng Instrument Co., Ltd., Hangzhou, China). RT-qPCR amplifications were measured in three biological replicates using a Bio-Rad CFX Connect System (Bio-Rad Laboratories, Inc., California, USA). The housekeeping gene, 16S rRNA, was chosen to normalize RNA amounts (internal control). The sequences coding for the 16S rRNA, *luxS* of the wild type (WT) SS-128 and the mutant strain Δ*luxS*/SS-128, were designed based on the *L. plantarum* WCFS1 gene sequences (GenBank, no. NC_004567.2, from https://www.ncbi.nlm.nih.gov/nuccore/NC_004567.2/, accessed on 10 April 2021). qPCR was performed with ChamQ SYBR Color qPCR Master Mix on a CFX Connect Real-Time PCR Detection System (Bio-Rad Laboratories, Inc., Hercules, CA, USA). The primer sequence is demonstrated in Table 1. Each experiment was repeated three times and analyzed using the 2^−^^∆ΔCT^ method.

### 2.3. Bacterial Growth Assay

SS-128 (wild type) and Δ*luxS*/SS-128 (*luxS* mutant type) were first propagated twice in MRS broth at 37 °C overnight. The 1% overnight cultures (18 h) were inoculated into a 5 mL fresh medium under aerobic conditions at 37 °C for 48 h. During the incubation, the bacterial density of the samples was determined every 2 h at 600 nm. Plate counting was used to determine the live cell number of the samples. The WT and mutant strains cultured for 14 h were collected by centrifugation and then were immediately frozen and stored at −80 °C.

### 2.4. Flow Cytometry Assay

The live/dead cells were determined by flow cytometry employing fluorescence after being stained with SYTO 9 and propidium iodide (PI). The counting method was constructed by Leonard and Bensch et al. [25,26], with some modifications. Cells (10^2^–10^3^ cells/μL) were incubated with SYTO 9 (5 μg/mL) and PI (5 μg/mL) for 3 min in dark. Flow cytometric analyses were carried out using a BD FACSVerse™ flow cytometry (BD Biosciences, San Jose, CA, USA). Data were analyzed by using FlowJo software 10.6.1 (Tree Star Inc., Ashland, OR, USA).

### 2.5. Determination of Bacteriostatic Ability

The agar diffusion assay was used to identify the bacteriostatic ability of cell-free supernatant (CFS) by Zhang and Li et al. [27,28]. A 15 mL aliquot of 0.4% soft agar with 2% bacterial culture medium was poured into a sterile Petri dish, and sterile Oxford cups (ϕ = 7.64 mm) were put on the surface of the medium. The Oxford cup was filled with the CFS of *L. plantarum* (200 μL). The inhibition zone could be measured after incubation at 28 °C for 24 h.

### 2.6. Transcriptome Analysis

RNA isolation and high-throughput RNA sequencing (RNA-Seq) were conducted by Oebiotech Corp. (Shanghai, China). Total RNA was extracted using a Total RNA Purification Kit (Sangon Biotech, Shanghai, China), followed by evaluating the RNA integrity on Agilent 2100 bioanalyzer (Agilent Technologies, Santa Clara, CA, USA). The libraries were sequenced on an Illumina sequencing platform (HiSeq 2500), and 150-bp/125-bp paired-end reads were generated. The gene expression profiles were analyzed based on reads per kilobase of transcript per million mapped read (RPKM) normalization. DESeq was used to standardize the counts of genes in each sample, and NB was used to test the difference significance of reads. Differential genes were screened according to the difference multiple and different significance test results. Finally, the GO and KEGG enrichment of differentially expressed genes were analyzed under the cutoff of *p*-value < 0.05 using the BLAST program.

### 2.7. Non-Targeted Metabolomic Analysis

#### 2.7.1. Sample Preparation

A total of 20 μL of 2-chloro-l-phenylalanine (0.3 mg/mL) dissolved in methanol as internal standard and methanol-water (4:1 = *v*/*v*) were added to each sample. Trichloromethane was added to each aliquot. The mixture was extracted in the ice water bath with ultrasonication for 20 min and then centrifugation (13,000 rpm, for 10 min at 4 °C). A total of 1 mL of supernatant was dried in a freeze concentration centrifugal dryer, sequentially redissolved in 25% methanol, and incubated at 4 °C for 2 min. After that, the mixtures were centrifuged again, using the same step as before. The supernatant of each tube was filtered to LC–MS and GC–MS for analysis. QC samples were prepared by mixing aliquots of all samples to be a pooled sample.

#### 2.7.2. Gas Chromatography/Mass Spectrometry (GC/MS) Analysis

The derivatized samples were analyzed on an Agilent 7890B gas chromatography system coupled to an Agilent 5977A MSD system (Agilent Technologies Inc., CA, USA). A DB-5MS fused-silica capillary column (30 m × 0.25 mm × 0.25 μm, Agilent J&W Scientific, Folsom, CA, USA) was utilized to separate the derivatives. Helium (>99.999%) was used as the carrier gas at a 1 mL/min constant flow rate through the column. The QCs were injected at regular intervals throughout the analytical run to provide a set of data from which repeatability could be assessed.

#### 2.7.3. Ultrahigh Performance Liquid Chromatography/Mass Spectrometry (UPLC/MS) Analysis

ACQUITY UPLC I-Class system (Waters Corporation Milford, Milford, MA, USA) coupled with VION IMS QTOF Mass spectrometer (Waters Corporation Milford, Milford, MA, USA) was used to analyze the metabolic profiling in both ESI positive and ESI negative ion modes. An ACQUITY UPLC BEH C18 column (1.7 μm, 2.1 × 100 mm) was employed in both positive and negative modes. Water and Acetonitrile/Methanol 2/3 (*v*/*v*), both containing 0.1% formic acid were used as mobile phases A and B, respectively. The injection volume was 1 μL. Data were collected in full scan mode (m/z ranges from 50 to 1000) combined with MSE mode, including two independent scans with different collision energies (CE) that were alternatively acquired during the running process.

#### 2.7.4. Data Preprocessing and Analysis

The acquired LC–MS raw data were analyzed by the progenesis QI software (Waters Corporation, Milford, CT, USA) to identify the metabolites. The raw data were converted to .abf format, followed by processing on the MD-DIAL software through LUG database (Untargeted database of GC–MS rom Lumingbio).

Principle component analysis (PCA) and (orthogonal) partial least-squares-discriminant analysis (O) PLS-DA were carried out to visualize the metabolic alterations among experimental groups after mean centering (Ctr) and Pareto variance (Par) scaling, respectively. Variable importance in the projection (VIP) ranks the overall contribution of each variable to the OPLS-DA model, and those variables with VIP > 1 are considered relevant for group discrimination. In this study, the default seven-round cross-validation was applied with one/seventh of the samples being excluded from the mathematical model in each round.

The differential metabolites were selected based on the combination of a statistically significant threshold of VIP values obtained from the OPLS-DA model and *p* values from a two-tailed Student’s t-test on the normalized peak areas, where metabolites with VIP values larger than 1.0 and *p* values less than 0.05 were considered as differential metabolites.

### 2.8. Statistical Analysis

All results were statistically analyzed with IBM SPSS Statistics version 19.0. Data were presented as means and standard deviations. One-way analysis of variance followed by Duncan’s post hoc test was used to compare the mean differences. A *p*-value < 0.05 was considered significant.

## 3. Results

### 3.1. Confirmation of luxS Gene Knockout

**Figure 1:**
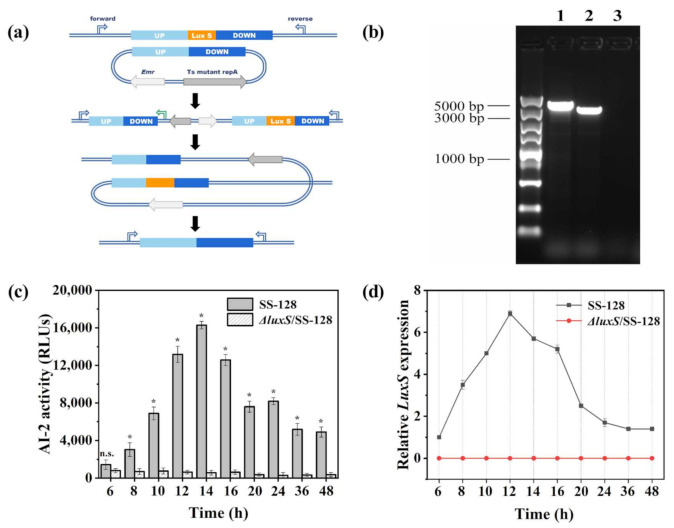
Two-step homologous recombination (**a**), PCR amplification (**b**), AI-2 activity (**c**), and transcription of the *luxS* gene (**d**) in SS-128 and Δ*luxS*/SS-128. All data points mean ± standard deviations (*n* = 3) with * denoting statistically significant differences (*p* < 0.05).

In order to confirm the function of *luxS* as a S-ribosylhomocysteine lyase, a knockout mutant of *luxS* was constructed. Gene *luxS* was successfully deleted by a temperature-sensitive plasmid pFED760 (Figure 1a). The PCR in the *luxS*-mutant generated a short 3224-bp DNA fragment (lane 1) as expected, while a normal 3701-bp DNA fragment (lane 2) was amplified in WT strain when using UD-f and UD-r primer pair in Table 1 (Figure 1b). From lane 3, it can be seen that the primers of *luxS* did not amplify the gene. The sequencing of PCR products also confirmed that the *luxS*-mutant was successfully achieved.

To determine the effect of *luxS* knockout on the AI-2 production ability of the strains, we monitored the AI-2 levels in the supernatants of *L. plantarum* SS-128 and Δ*luxS*/SS-128 by bioluminescence assay using the *V. harveyi* BB170 strain. Lack of the *luxS* gene, which encodes an enzyme involved in methionine metabolism, ultimately leads to the loss of AI-2. As shown in Figure 1c, the accumulation concentration of AI-2 of *L. plantarum* SS-128 was at its highest in the late exponential phase and diminished slowly in the stationary phase. It was agreed with the results of Amandeep et al. [29], which reported that AI-2 activity increased till the late exponential phase. Moreover, the results demonstrated that AI-2 activities of SS-128 were significantly increased at 14 h compared to those of Δ*luxS*/SS-128 combined with transcript-level expression analysis (Figure 1d). These data suggested that the deletion of the *luxS* contributed to a disability in synthesizing AI-2.

### 3.2. Growth Characteristics of L. plantarum SS-128 and ΔluxS/SS-128

**Figure 2:**
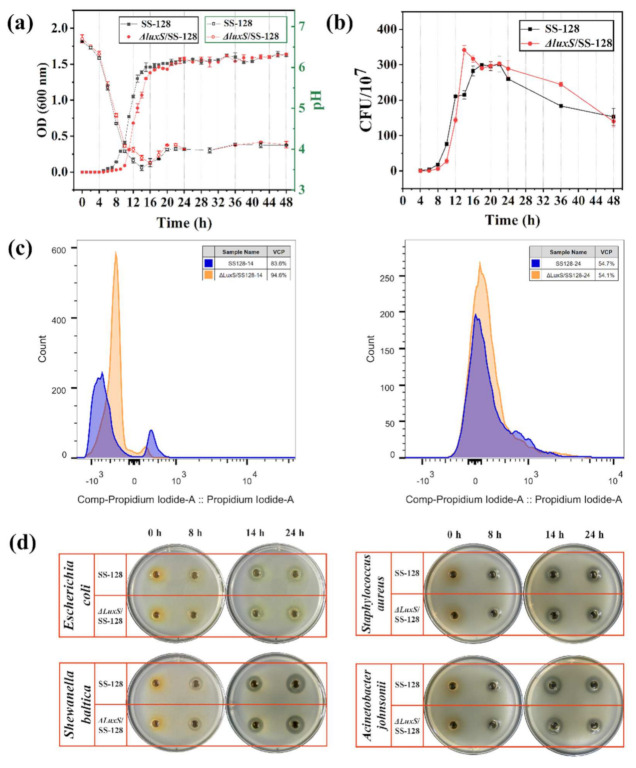
Cell density (**a**), live cell number (**b**), VCP of *L. plantarum* by flow cytometry (**c**), and images of inhibition zone of SS-128 and Δ*luxS*/SS-128 against *E. coli*, *S. baltica*, *S. aureus,* and *A. johnsonii* (**d**). All data points mean ± standard deviations (*n* = 3).

The behaviors of *L. plantarum* exposed to 37 °C were summarized in Figure 2. Results in Figure 2a showed that the tested SS-128 started to grow exponentially earlier compared with Δ*luxS*/SS-128. The pH of SS-128 reached its lowest value (3.54) after 14 h of cultivation, then began to increase, and the time was 2 h earlier than that of defective strain. These properties differ from the growth rate of *L. reuteri luxS* mutant [19]. This might be account for the *cysK* enzyme in *L. plantarum*. Gu et al. reported that the exogenous synthetic AI-2 led to different growth feedback on physiological behaviors of *Enterococcus faecium* and *Lactobacillus fermentum* in vitro [15,16]. However, the live cell number of the Δ*luxS*/SS-128 was significantly higher than that of the SS-128 at the late exponential phase (12–18 h) (Figure 2b). It is speculated that the proportion of viable bacteria in Δ*luxS*/SS-128 was higher than that in the SS-128 within 12–18 h.

For determining the viable cell proportion (VCP) of *L. plantarum*, the SYTO 9 and PI staining was used to analyze the membrane integrity by flow cytometry. The results of double-staining on *L. plantarum* (Figure 2c) showed the live/dead cells, respectively. The VCP was 83.6% in SS-128 and 94.6% in Δ*luxS*/SS-128 at 14 h, and no apparent difference in the proportion of viable bacteria at 24 h was shown in Figure 2c (*p* < 0.05). The results demonstrated that the VCP in Δ*luxS*/SS-128 was higher than that in SS-128 during the late exponential phase. *L. plantarum* proliferates by binary fission, in which the cell must double its mass, replicate its DNA and divide equally to produce two daughter cells [30]. Based on this way of splitting and the adequate nutrition, the higher total number of living bacteria should exhibit an exponential increase with the normal proliferation rate of normal cells. However, the live cell number of the *luxS* mutant did not increase many times as much as that of the wild strain at the late exponential phase, contrary to the normal rate of the cell proliferation. Empirical evidence suggests that *E. coli* and *Schizosaccharomyces pombe*, all copied by binary division, avoid division by prolonging senescence when conditions are unfavorable [31,32]. Therefore, we hypothesized that the *luxS* gene might regulate cell proliferation, thereby affecting cell growth.

### 3.3. In Vitro Bacteriostatic Effect of L. plantarum SS-128 and ΔluxS/SS-128

LAB have great potential in hindering the activity of pathogenic and spoilage bacteria in food systems by producing bacteriostatic compounds mainly composed of organic acids [4,33]. Thus, we compared the bacteriostatic ability of cell-free supernatant against *E. coli*, *Shewanella baltica*, *Staphylococcus aureus* and *Acinetobacter johnsonii* of *L. plantarum* SS-128 and Δ*luxS*/SS-128 (Figure 2d). *E. coli* and *S. aureus* are common food spoilage and pathogenic microorganisms [34,35,36], while *A. johnsonii* and *P. fluorescens* are the main spoilage potential organism during the iced storage of aquatic products [37,38], which may pose considerable risks to the health of consumers. The activity was evaluated by Oxford cup tests with the sample from that of the CFS; the MRS broth without *L. plantarum* as the control. The inhibitory zone diameter of each sample was measured with calipers and summarized in Table S1. As shown in Figure 2d, the control group showed no bacteriostatic ring, indicating that MRS broth has no bacteriostatic ability against the food-borne spoilage and pathogenic bacteria. In contrast, SS-128 exhibited a clear antibacterial ring around the bacteria and was larger than that of Δ*luxS*/SS-128 after 14 h. In particular, the CFS of SS-128 collected at 24 h showed significant antibacterial activity against *S. baltica* (16.41 mm) compared with Δ*luxS*/SS-128 (15.18 mm) (*p* < 0.05). According to the above results, the metabolites of SS-128 had stronger bacteriostatic ability.

### 3.4. The Organic Acids Production of L. plantarum SS-128 and ΔluxS/SS-128

**Figure 3:**
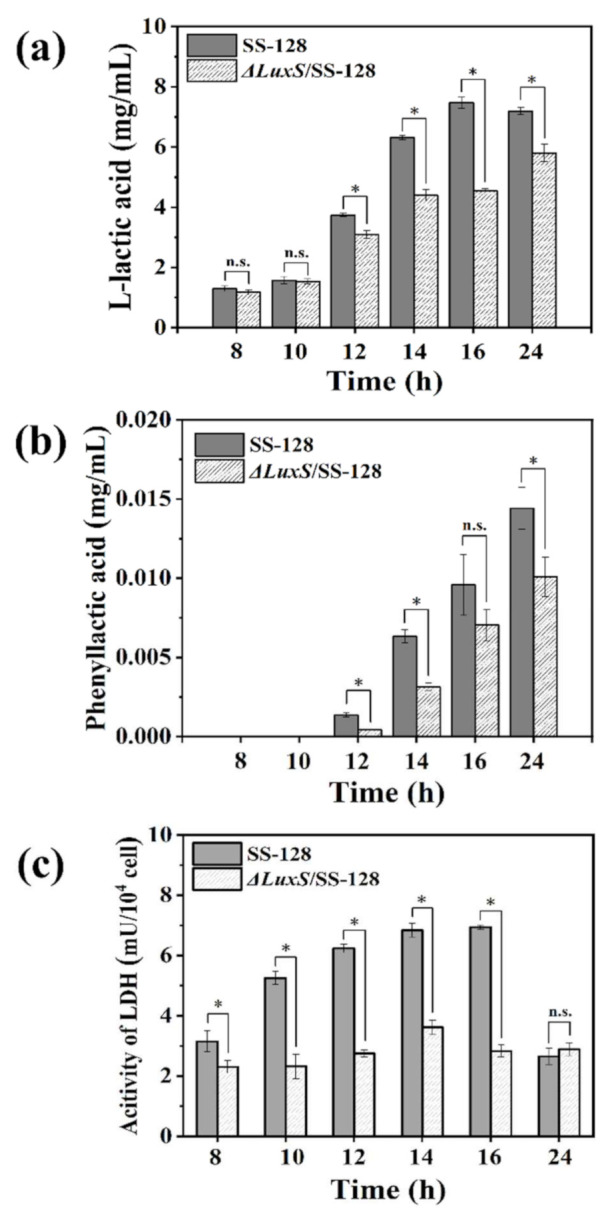
Extracellular concentrations of L-lactic acid (**a**) and PLA (**b**) detected by HPLC in SS-128 and Δ*luxS*/SS-128; LDH activity (**c**). All data points mean ± standard deviations (*n* = 3) with * denoting statistically significant differences (*p* < 0.05); n.s.: not significant.

To further explore the regulation of the *luxS* gene on the main bacteriostatic metabolites (organic acids) produced by *L. plantarum*, the organic acids production in *L. plantarum* SS-128 and Δ*luxS*/SS-128 was measured. The data suggested that the organic acid production of *L. plantarum* SS-128 was significantly decreased upon knockout of the *luxS* gene (Figure 3a,b). The lactic acid production of *L. plantarum* SS-128 was 20.96% to 64.12% higher than that of the *luxS* mutant type in 12–16 h (*p* < 0.05) (Figure 3a). QS is widely believed to regulate the metabolism of bacteria (e.g., *Pseudomonas aeruginosa* and *S. baltica*) [39,40]. Similar results were observed for PLA production in 12–14 h. These results indicated that the production of lactic acid and PLA in *L. plantarum* might be related to the regulation of AI-2/LuxS system. In our study, a lower number of viable bacteria and stronger bacteriostatic ability persisted in SS-128 compared with Δ*luxS*/SS-128 after 14 h, which could further confirm the regulation of *luxS* on the growth characteristics and metabolism of *L. plantarum*.

Lactate dehydrogenase (LDH), widely distributed in diverse sources including LAB, catalyzes the reduction of pyruvate to lactic acid. It can also catalyze the conversion of phenylpyruvic acid (PPA) to PLA due to its broad substrate specificity [41]. Therefore, the determination of LDH activity could reflect the effect of *luxS* gene on the acid production capacity of *L. plantarum*. Figure 3c shows the LDH activity in *L. plantarum* evaluated after 8, 10, 12, 14, 16, and 24 h of cultivation. Compared with Δ*luxS*/SS-128 (2.32 mU/10^4^ cell), LDH activity was significantly increased in SS-128 after 10 h (4.25 mU/10^4^ cell) (*p* < 0.05). During the exponential growth period, the LDH activity of SS-128 remained stable and significantly higher than that of Δ*luxS*/SS-128 (*p* < 0.05).

### 3.5. Transcriptomics Analyses between L. plantarum SS-128 and the ΔluxS/SS-128

**Figure 4:**
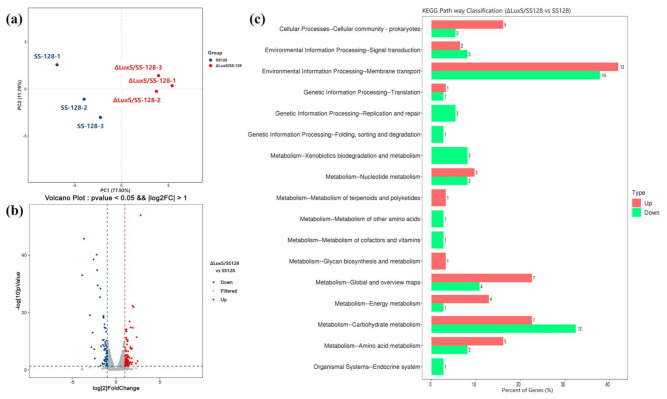
PCA analysis (**a**), volcano plot (**b**), and KEGG pathway of DEGs at level 2, (**c**) (Δ*luxS*/SS-128 vs. SS-128).

The *luxS* gene is involved in QS, which has been shown to regulate critical physiological traits and various adaptive processes in different bacteria [42,43]. Therefore, probing the relationship between AI-/LuxS-mediated QS and the cell transcriptional expression levels is the premise to influence bacterial metabolites accumulation. Based on this, the expression profile of *L. plantarum* without *luxS* gene was discerning, and transcriptome analysis was performed on wild type and mutant type. Overall, 22.92 and 22.83 million reads of SS-128 and Δ*luxS*/SS-128 were used for further analysis, respectively, and 94.06–94.48% of the total reads mapped to the reference genome, among which 66.10–76.02% were coding sequence (CDS) mapped reads (Table S2). There were significant differences between Δ*luxS*/SS-128 and SS-128 in PCA model (Figure 4a). The differences between the first and the second principal component were 77.93% and 11.78%, respectively. A total of 157 genes were identified in Δ*luxS*/SS-128 from uniquely matched reads data compared to SS-128 expressing at different levels (*p* < 0.05) (Figure 4b).

The 157 differentially expressed genes (DEGs) were subsequently applied to functional analysis in Kyoto Encyclopedia of Genes and Genomes (KEGG) pathway functional enrichment. As shown in Figure 4c, KEGG enrichment analysis found that some pathways were remarkably changed after the *luxS* gene knocked out in the histogram. These pathways included “Membrane transport”, “Carbohydrate metabolism”, and “Amino acid metabolism”. Our data indicated that, owing to the loss of the *luxS* gene, a series of transport-related pathways were adjusted, and the pathways involved in energy metabolism were also changed in the process of cell growth. The identified DEGs were combined with functional analysis results, such as KEGG annotations based on previous reports. These genes were considered the most critical genes related to cell growth and accumulation of metabolites, mainly involved in PTS, tricarboxylic acid (TCA), and methionine cycle (Table 2).

### 3.6. Metabolomics Analyses between L. plantarum SS-128 and the ΔluxS/SS-128

**Figure 5:**
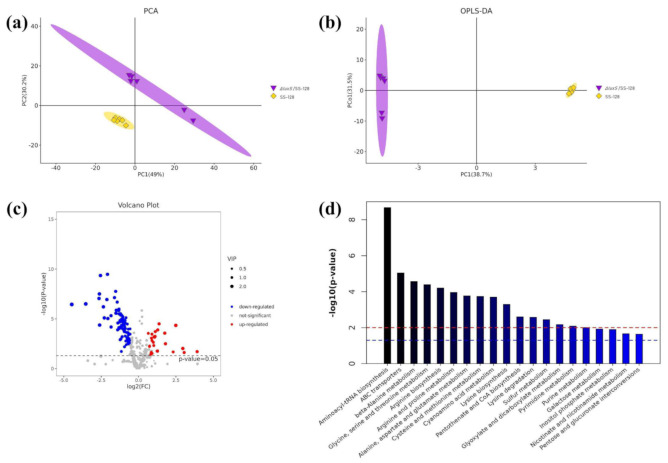
Changes in the metabolite contents compared the *luxS* mutant group with the control by GC–MS. The PCA (**a**), OPLS-DA (**b**), volcano plot (**c**), and metabolic pathway enrichment map-Top 20 (**d**) between Δ*luxS*/SS-128 and SS-128 under the cut off of *p* < 0.05 (Δ*luxS*/SS-128 vs. SS-128). The red line and blue line in (**d**) indicate *p* = 0.01 and 0.05, respectively.

Except for transcriptome sequencing analysis, the untargeted GC–MS and LC–MS metabolomics analyses were used to further explore the adaptation of metabolic processes in *L. plantarum* SS-128 response to the *luxS* gene. The total number of metabolites detected in samples was 357, 2316, and 1330 for GC–MS, LC–MS positive ion mode, and negative ion mode, respectively. The tested samples were apparently separated from the wild type and mutant type in Figure 5a and Figure S1a. Furthermore, the first principal components (PC) were clearly separated by the OPLS-DA model, corresponding a variation between groups of 38.7% (GC–MS) and 54.7% (LC–MS). These data indicated that the cell metabolism was changed significantly owing to the knocked out of the *luxS* gene (Figure 5b and Figure S1b). The OPLS-DA model screened out differentially expressed metabolites in the two groups. The structure identities of 110 metabolites screened by GC–MS were assigned, among which 26 metabolites were significantly upregulated and 84 metabolites were significantly downregulated (*p* < 0.05) (Figure 5c). LC–MS identified a total of 464 differentially expressed metabolites. Meanwhile, there were 17 metabolites substantial decreased and 33 metabolites increased in the first 50 metabolites that exhibited significant differences (*p* < 0.05) (Figure S1c).

To investigate the metabolic pathways involved, KEGG pathway analysis was performed. Among the top-20 metabolic pathway enrichment map, the differentially expressed metabolites (DEMs) in mutant type compared with WT strains were mainly enriched on aminoacyl-rRNA biosynthesis, ABC transporters, amino acid metabolism, pyrimidine metabolism and purine metabolism (Figure 5d). Based on the above, the key differential metabolites involved in the enriched pathways were listed in Table 3.

## 4. Discussion

Since the above results showed that AI-2/LuxS positively regulated the bacteriostatic activity and growth of *L. plantarum* SS-128, to explore the correlation between two regulatory networks, the metabolome and transcriptome data were subjected to integrated analysis. The results suggest that the knockout of *luxS* gene resulted in the overall downregulation of methionine and cysteine cycling pathways. By comparing the Δ*luxS*/SS-128 with the SS-128, the metabolites (SAM, L-cystathionine, O-Acetyl-L-Serine, et al.) and genes (*luxS* and *cysK*) showed a lower expression in the mutant strain. Due to the disruption of the methionine cycle, L-homocysteine cannot be replenished-resulting in the consumption of a large SAM as methyl provider, and eventually, the dynamic cycle is disrupted. It is worth noting that it is converted to S-adenosine homocysteine (SAH) when methyl groups are transferred from SAM to nucleic acids, proteins, or other compounds via a SAM-dependent methyltransferase [44]. Therefore, SAM participated in DNA and protein methylation as a major methyl donor for methylation in living cells [45]. Thus, it can be inferred that *luxS* in the methionine cycle is involved in the regulation of methylation of DNA and other substances and cysteine metabolic pathways.

Bacteria, yeast, and mammalian cells are all dependent on an adequate supply of purines and pyrimidines to maintain proliferation. The proliferation of LAB requires a deoxyriboside bound to purine and pyrimidine bases [46]. As shown in Table 3, the DEMs, such as hypoxanthine, uracil, and riboflavin, were significantly increased by 5.61, 2.59, and 2.22 times, respectively. Therefore, the differential purine metabolites may be the response of the *luxS* mutant strain to changes in cellular metabolism, which is consistent with previous similar studies. It was reported that purine and purine precursors are related to cell growth under stress conditions [47,48,49]. Uracil dramatically affected *L. plantarum* growth incubated in ordinary air, and uracil sensitivity in aerobiosis was found in *L. plantarum* [50]. The accumulation of metabolites in the nucleotide synthesis pathway in cells reflects the delay of nucleotide metabolism in cell proliferation, further confirming the regulation of *luxS* on cell proliferation.

**Figure 6:**
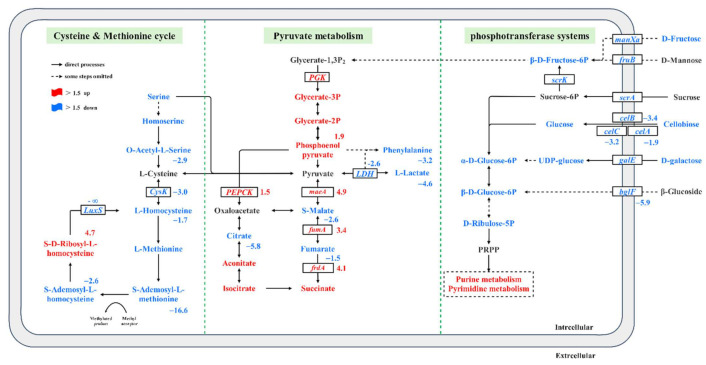
Changes of the expression levels of genes and metabolisms involved in *L. plantarum* (Δ*luxS*/SS-128 vs. SS-128). Pathways were constructed based on information provided in the KEGG database and previous studies. Microcompartments are depicted by dotted green lines.

A significant increase in SRH was observed due to the interruption of activated methyl cycle (AMC) and store depletion of L-homocysteine. Intracellular L-cysteine levels have properly been regulated for proper cell growth [51]. As shown in Figure 6, L-cysteine was maintained only by the pyruvate, while the contents of metabolites in other related metabolic pathways were significantly decreased. The phenomenon is similar to the “anaplerotic sequences” described by Hans Kornberg in Biochemistry that some key intermediates are removed during the growth process, other related metabolites are used for the net production of the intermediate [52]. This is consistent with previous studies in which cysteine-deficient cells exhibited over 100-fold increased oxidative stress compared with the cysteine-added cells and was associated with protective mechanisms of cell growth [53]. Since pyruvate is a major metabolic junction linking carbohydrate or amino acid utilization to energy generation and biosynthetic pathways, the content of key intermediate metabolites requires timely replenishment through auxiliary reactions [54,55]. In response to the interruption of the methionine cycle, glycolysis and TCA cycle were upregulated to “maintain” the ATP for energy and NADPH for reducing power. DEGs related to pyruvate metabolism and TCA cycle, such as *pckA*, *ME2*, *fumA,* and *frdA,* were upregulated in Δ*luxS*/SS-128 compared to the control. Whereas operated by the “anaplerotic sequences”, L-lactic, PLA, malate and fumarate synthesis were downregulated to maintain the overall rate of pyruvate, oxaloacetate, and phosphoenolpyruvate synthesis.

The primary fermentation metabolite of LAB is lactic acid, which results in an environment of the lower pH which generally limits the growth of pathogenic and spoilage microbes [3]. The solubility of undissociated lactic acid within the cytoplasm membrane and the insolubility of dissociated lactic acid may affect the pH gradient across the membrane and reduce the available energy for cell growth [56]. As shown in Figure 6 and Table 3, while succinic acid was accumulated in the TCA cycle, the lactic acid and PLA produced by fermentation of the Δ*luxS*/SS-128 was significantly lower than that of the SS-128. This conclusion was consistent with the result of lactic acid and PLA production by extracellular accumulation of SS-128 and Δ*luxS*/SS-128. Yang et al. reported that exogenous DPD could promote the growth and the production of PLA of *L. Plantarum* AB-1 cells [57]. Similarly, our data suggest that the lactic acid and PLA production of *L. plantarum* SS-128 was regulated by the AI-2/LuxS system. The crucial enzyme in lactic acid fermentation is LDH, which is mainly responsible for catalyzing the reversible reduction of pyruvate to lactic acid [58]. Recently, LDH has also been shown to be the main enzyme catalyzing the PPA to PLA, which is a broad-spectrum antibacterial compound [59,60]. The significant reduction of *ldh* of defective strains in transcriptomics results was also consistent with our previous experimental results (*p* < 0.05) (Figure 3c). The results indicated that the pyruvate production was maintained by increasing the directly related anabolism of the Δ*luxS*/SS-128, followed by a reduction in directly related catabolism. Thus, downregulation of the *ldh* reduces the production of the organic acid.

In addition, our results showed that the yield of malic acid, fumaric acid and citric acid in Δ*luxS*/SS-128 were also significantly downregulated (*p* < 0.05). The production of organic acids is undoubtedly the decisive factor to prolong the shelf life and safety of products by LAB, which was an important indicator to reflect the biocontrol ability. The significant decrease of organic acid production confirmed the regulation effect of *luxS* on the bacteriostatic ability of LAB.

Unlike pyruvate metabolism to output energy in the form of ATP, PTS is an ATP-dependent mechanism for absorbing sugars. It is dominant among the three mechanisms (PTS, secondary carriers and ABC transporters) of LAB, accounting for about 52%. It provides an essential carbon source to support lipid production and the biosynthesis of nucleotides and non-essential amino acids (NEAAs). Due to the various carbohydrate sources supplemented in MRS medium, the result of combined omics analysis could reflect the difference in carbohydrate transport capacity of SS-128 compared with Δ*luxS*/SS-128. More than 200 unigenes were involved in carbohydrate metabolism during the growth of *L. plantarum*, and 36 of these unigenes were involved in membrane transporter. Twenty-seven of these unigenes were DEGs in the PTS, and 17 of them in KEGG enrich top 20 (Figure 4) were summarized in Table 2. The results suggested that most of the DEGs associated with PTS were significantly downregulated (*p* < 0.05). Genes *celA*, *celB,* and *celC* encoding cellobiose transport system permease are responsible for the carbohydrates utilization in Δ*luxS*/SS-128, and significantly downregulated 1.92-, 3.36-, and 3.19-fold compared to SS-128. The *bglF* was downregulated 5.93-fold change in response to stimulating growth. Normal cells often increase the uptake of glucose to provide the significant carbon source for the production of lipids, nucleotides, and non-essential amino acids [61,62]. Therefore, lower transcript levels of genes encoding specific carbohydrate PTS components indicate that Δ*luxS*/SS-128 may only be able to uptake and utilize the limited range of energy substances, as part of a defense mechanism against the genetic mutations, and this could explain their less efficient for growth and lower biocontrol ability.

## 5. Conclusions

In summary, the comparative multiomic analyses demonstrated that the higher pyruvate metabolic efficiency and energy input, followed by higher LDH level and metabolite overflow in SS-128, resulted in stronger bacteriostatic ability compared to that of Δ*luxS*/SS-128. These results suggest that the AI-2/LuxS could positively regulate the bacteriostatic activity and growth of *L. plantarum* SS-128. Our data indicated that *L. plantarum* as bio-preservatives was mediated by AI-2/luxS targeting to improve food safety, but further studies are needed to explore the strategies of enhancing positive regulation.

## Figures and Tables

**Table 1:** Primer sequences.

Gene Name	Oligonucleotide Sequence (5′-3′)	
Up	Forward	TATCCC ACTACCTGAA ACTCG
	Reverse	GCACCACCATTACTTTTTATATTGTAGCACATTGCCCGTTA
Down	Forward	TAACGGGCAATGTGCATACAATATAAAAAGTAATGGTGGTGC
	Reverse	GCTGGTGCTTCGTAAACTTCC
16S rRNA	Forward	CGTAGGTGGCAAGCGTTGTCC
	Reverse	CGCCTTCGCCACTGGTGTTC
LuxS	Forward	CGGATGGATGGCGTGATTGACTG
	Reverse	CTTAGCAACTTCAACGGTGTCATGTTC
UD	Forward	GTTCTGCACGGACGCTATCT
	Reverse	ATTAACTTGCGTTGGTAGGC

**Table 2:** DEGs involved in PTS, pyruvate metabolism and quorum sensing.

Locus Tag	Entry	Gene Name (ID)	Definition	Fold Change	*p*-Value
PTS	K02793	manXa	mannose PTS system EIIA component	1.56 ↓	1.12 × 10^4^
	K02768	fruB	fructose PTS system EIIA component	1.68 ↓	2.14 × 10^6^
	K02810	scrA, ptsS	sucrose PTS system EIIBCA component	1.54 ↓	8.15 × 10^3^
	K02761	celB, chbC	cellobiose PTS system EIIC component	3.36 ↓ *	2.21 × 10^4^
	K02760	celA, chbB	cellobiose PTS system EIIB component	1.92 ↓	6.99 × 10^4^
	K02759	celC, chbA	cellobiose PTS system EIIA component	3.19 ↓*	4.32 × 10^4^
	K02773	gatA, sgcA	galactitol PTS system EIIA component	1.88 ↓	2.76 × 10^4^
	K02774	gatB, sgcB	galactitol PTS system EIIB component	1.71 ↓	1.46 × 10^2^
	K02755	bglF	beta-glucoside PTS system EIIA component	5.93 ↓ *	3.28 × 10^9^
	K02798	cmtB	mannitol PTS system EIIA component	1.59 ↓	1.49 × 10^5^
		LP_RS14755	PTS system EIIC component	1.74 ↓	1.79 × 10^2^
		LP_RS12340	PTS system EIIA component	1.67 ↓	3.06 × 10^2^
		LP_RS12650	PTS system EIIB component	1.52 ↓	4.89 × 10^2^
		LP_RS12655	PTS system EIIC component	2.33 ↓ *	9.25× 10^10^
		LP_RS13505	beta-glucoside PTS system EIIBCA component	1.54 ↓	8.15× 10^3^
		LP_RS14845	PTS system EIIB component	1.71 ↓	1.46 × 10^2^
		LP_RS14850	PTS system EIIA component	1.88 ↓	2.76 × 10^4^
Pyruvate metabolism	K00927	PGK, pgk	phosphoglycerate kinase	1.52 ↑	1.85 × 10^5^
	K00016	ldh	L-lactate dehydrogenase	2.58 ↓ *	1.03 × 10^2^
	K01610	pckA, PEPCK	phosphoenolpyruvate carboxykinase (ATP)	1.51 ↑	3.95 × 10^2^
	K00027	ME, maeA	malate dehydrogenase	4.87 ↑ *	5.68 × 10^3^
	K01676	fumA, fumB	fumarate hydratase, class I	3.39 ↑ *	1.10 × 10^4^
	K00244	frdA	fumarate reductase flavoprotein subunit	4.08 ↑ *	3.14 × 10^2^
	K01744	aspA	aspartate ammonia-lyase	1.70 ↑	1.99 × 10^7^
	K01939	purA, ADSS	adenylosuccinate synthase	1.55 ↑	2.25 × 10^2^
	K01512	acyP	acylphosphatase	1.70 ↑	2.95 × 10^5^
Methionine cycle	K07173	luxS	S-ribosylhomocysteine lyase		2.53 × 10^4^
	K01738	cysK	cysteine synthase	2.95 ↓	5.85 × 10^10^
	K01999	livK	branched-chain amino acid transport system substrate-binding protein	2.01 ↓ *	2.75 × 10^2^

Fold change was Δ*luxS*/SS-128 vs. SS-128. ↑ and ↓ represented up and down regulation, respectively. * fold change was significantly higher than 2 (*p* < 0.05).

**Table 3:** Differential metabolites involving PTS, nucleotide synthesis, pyruvate metabolism, cysteine and methionine metabolism.

Metabolites	KEGG	KEGG Annotation	Dataclass	Formula	VIP	*p*-Value	FC
2′-Deoxyguanosine 5′-monophosphate	C00362	Purine metabolism	LC	C10H14N5O7P	1.12	3.46 × 10^8^	1.53 ↑
Deoxyguanosine	C00330	Purine metabolism	LC	C10H13N5O4	1.26	2.38 × 10^6^	1.84 ↑
Guanine	C00242	Purine metabolism	GC	C5H5N5O	1.38	6.32 × 10^4^	2.05 ↑ *
Hypoxanthine	C00262	Purine metabolism	GC	C5H4N4O2	2.27	4.52 × 10^5^	5.61 ↑ *
Xanthine	C00385	Purine metabolism	LC	C5H4N4O2	1.14	1.08 × 10^7^	2.21 ↑ *
Cytosine	C00380	Pyrimidine metabolism	GC	C4H5N3O	1.43	5.73 × 10^4^	2.15 ↑ *
dCMP	C00239	Pyrimidine metabolism	LC	C9H14N3O7P	1.62	1.58 × 10^4^	1.62 ↑
dTMP	C00364	Pyrimidine metabolism	LC	C10H15N2O8P	5.82	1.20 × 10^7^	1.73 ↑
Pseudouridine 5′-phosphate	C01168	Pyrimidine metabolism	LC	C9H13N2O9P	4.18	6.41 × 10^5^	1.15 ↑
Uracil	C00106	Pyrimidine metabolism	GC	C4H4N2O2	1.67	3.17 × 10^5^	2.59 ↑ *
Uridine	C00299	Pyrimidine metabolism	GC	C9H12N2O6	1.29	3.43 × 10^4^	1.86 ↑
Uridine 5′-monophosphate	C00105	Pyrimidine metabolism	LC	C9H13N2O9P	2.20	5.38 × 10^4^	1.42 ↑
Orotidine	C01103	Pyrimidine metabolism	LC	C10H12N2O8	1.42	7.33 × 10^7^	4.61 ↓ *
L-glutamine	C00064	Purine metabolism pyrimidine metabolism	GC	C5H10N2O3	3.13	3.58 × 10^7^	22.12 ↓ *
Flavin Mononucleotide	C00061	Oxidative phosphorylation Riboflavin metabolism	LC	C17H21N4O9P	1.04	4.29 × 10^3^	1.66 ↑
Riboflavin	C00255	ABC transporters Riboflavin metabolism	LC	C17H20N4O6	1.23	3.40 × 10^3^	2.22 ↑ *
Flavin adenine dinucleotide	C00016	Riboflavin metabolism	LC	C27H33N9O15P2	2.96	2.77 × 10^10^	1.64 ↑
D-Glycerate 2-phosphate	C00631	Glycolysis/Gluconeogenesis	LC	C3H7O7P	1.81	2.85 × 10^7^	1.84 ↑
D-Glycerate 3-phosphate	C00197	Glycolysis/Gluconeogenesis	LC	C3H7O7P	1.10	9.00 × 10^8^	1.79 ↑
Phosphoenolpyruvic acid	C00074	Glycolysis/Gluconeogenesis Citrate cycle (TCA cycle)	LC	C3H5O6P	1.86	5.37 × 10^8^	1.91 ↑
Isocitric acid	C00311	Citrate cycle (TCA cycle)	GC	C6H8O7	1.22	2.09 × 10^2^	4.97 ↑ *
Succinic acid	C00042	Citrate cycle (TCA cycle)	GC	C4H6O4	1.47	1.91 × 10^4^	2.17 ↑ *
L-aspartate	C00049	Cysteine and methionine metabolism	GC	C4H7NO4	1.29	4.77 × 10^3^	2.02 ↑ *
L-glutamate	C00025	Alanine, aspartate and glutamate metabolism	LC	C5H9NO4	8.24	1.44 × 10^5^	1.52 ↑
N-acetyl-glutamate	C01250	Arginine biosynthesis	GC	C7H11NO4	1.84	2.72 × 10^4^	3.37 ↑ *
Glutathione (GSH)	C00051	Cysteine and methionine metabolism | ABC transporters	GC	C10H17N3O6S	1.25	8.57 × 10^4^	1.82 ↑
L-gamma-glutamyl-L-valine	C03740	Glutathione metabolism	LC	C10H18N2O5	1.34	3.31 × 10^6^	1.34 ↑
Malic acid	C00149	Citrate cycle (TCA cycle)	GC	C4H6O5	1.13	2.28 × 10^4^	2.62 ↓
Fumaric acid	C00122	Citrate cycle (TCA cycle)	GC	C4H4O4	1.49	3.51 × 10^3^	1.52 ↓
L-lactic acid	C00186	Pyruvate metabolism	GC	C3H6O3	2.05	5.33 × 10^4^	4.56 ↓
L-Phenylalanine	C00079	Phenylalanine metabolism	LC	C9H11NO2	5.94	2.58 × 10^4^	3.24 ↓
Phenyllactic acid	C05607	Phenylalanine metabolism	LC	C9H10O3	1.15	8.02 × 10^8^	1.61 ↓
Aconitic acid	C00417	Citrate cycle (TCA cycle)	GC	C6H6O6	1.19	2.58 × 10^4^	1.54 ↑
Citric acid	C00158	Citrate cycle (TCA cycle)	GC	C6H8O7	2.62	3.81 × 10^6^	5.75 ↓ *
L-methionine	C00073	Cysteine and methionine metabolism	GC	C5H11NO2S	1.17	1.14 × 10^3^	1.69 ↓
S-adenosyl-L-methionine (SAM)	C00019	Cysteine and methionine metabolism	LC	C15H22N6O5S	1.81	1.49 × 10^10^	16.66 ↓ *
S-ribosyl-L-homocysteine (SRH)	C03539	Cysteine and methionine metabolism	LC	C9H17NO6S	5.76	1.00 × 10^8^	4.71 ↑ *
L-cystathionine	C02291	Cysteine and methionine metabolism	GC	C7H14N2O4S	2.40	9.01 × 10^8^	6.85 ↓ *
L-homocysteine	C00155	Cysteine and methionine metabolism	GC	C4H9NO2S	2.03	1.44 × 10^5^	1.73 ↓
S-adenosyl-L-homocysteine (SAH)	C00021	Cysteine and methionine metabolism	GC	C14H20N6O5S	2.92	2.72 × 10^4^	2.58 ↓
Serine	C00065	Cysteine and methionine metabolism	GC	C3H7NO3	1.58	6.93 × 10^5^	0.42 ↓
Homoserine	C00263	Cysteine and methionine metabolism	GC	C4H9NO3	1.76	1.39 × 10^6^	2.80 ↓ *
O-acetyl-L-serine	C00979	Cysteine and methionine metabolism	GC	C5H9NO4	1.51	1.02 × 10^5^	2.89 ↓ *
Creatine	C00300	Glycine, serine and threonine metabolism	LC	C4H9N3O2	2.01	1.23 × 10^8^	0.65 ↓
D-fructose 2,6-bisphosphate	C00665	Fructose and mannose metabolism	GC	C6H14O12P2	1.51	1.04 × 10^3^	2.27 ↓ *
D-fructose	C02336	Phosphotransferase system (PTS) Fructose and mannose metabolism	GC	C6H12O6	1.74	2.06 × 10^7^	2.70 ↓ *
Cellobiose	C00185	Phosphotransferase system (PTS)	GC	C12H22O11	1.73	1.40 × 10^6^	1.59 ↓
Galactose	C00984	Galactose metabolism Phosphotransferase system (PTS)	GC	C6H12O6	1.48	5.98 × 10^6^	2.08 ↓ *
Glucose-6-phosphate	C00092	Phosphotransferase system (PTS)	GC	C6H13O9P	1.35	1.41 × 10^4^	1.93 ↓
D-ribulose 5-phosphate	C00199	Pentose phosphate pathway	GC	C5H11O8P	1.09	3.31 × 10^3^	1.61 ↓
UDP-glucose	C00029	Pyrimidine metabolism Pentose and glucuronate	LC	C15H24N2O17P2	1.53	3.97 × 10^5^	3.80 ↓ *
D-glucose	C00031	Glycolysis/Gluconeogenesis Phosphotransferase system (PTS)	LC	C6H12O6	2.04	2.79 × 10^5^	2.33 ↓ *

Fold change was Δ*luxS*/SS-128 vs. SS-128. ↑ and ↓ represented up and down regulation, respectively. * fold change was significantly higher than 2 (*p* < 0.05).

## Data Availability

Not applicable.

## Supplementary Materials

The following supporting information can be downloaded at: https://www.mdpi.com/article/10.3390/foods11050638/s1, Figure S1: Changes in the metabolite contents compared the *luxS* mutant group with the control by LC-MS; Table S1: The mean inhibition zone diameter (mm) of CFS of *L. plantarum* against the *E. coli*, *S. baltica*, *S. aureus* and *A. johnsonii*; Table S2: Comparison of statistical results and reference genome.
