# Supplementary material for: Dissecting of the AI-2/LuxS Mediated Growth Characteristics and Bacteriostatic Ability of Lactiplantibacillus plantarum SS-128 by Integration of Transcriptomics and Metabolomics
Source: Foods. 2022 Feb 22;11(5):638. doi: 10.3390/foods11050638 (PMC8909743; doi:10.3390/foods11050638)
Supplement: Supplementary file 1 [file foods-11-00638-s001.zip › foods-1592924-supplementary.pdf]

foods

Supplementary Materials

Dissecting of the AI-2/LuxS Mediated Growth Characteristics and Bacteriostatic Ability of *Lactiplantibacillus plantarum* SS-128 by Integration of Transcriptomics and Metabolomics

Yilin Qian, Yuan Li, Tengteng Xu, Huijuan Zhao, Mingyong Zeng and Zunying Liu *

College of Food Science and Engineering, Ocean University of China, Qingdao 266003, China; qianyl92@outlook.com (Y.Q.); liyuan@ouc.edu.cn (Y.L.); xtteng924@163.com (T.X.); zhj13583036006@163.com (H.Z.); mingyz@ouc.edu.cn (M.Z.)

* Correspondence: liuzunying@ouc.edu.cn; Tel./Fax: +86-532-8203-2400

Table S1. The mean inhibition zone diameter (mm) of CFS of *L. plantarum* against the *E. coli*, *S. baltica*, *S. aureus* and *A. johnsonii*.

Time (h)		*E. coli*	*S. aureus*	*S. baltica*	*A. johnsonii*
8	SS-128	9.67±0.07^{e}	9.79±0.11^{c}	10.41±0.08^{e}	9.24±0.13^{e}
	ΔLuxS/SS-128	9.08±0.02^{f}	9.84±0.08^{c}	10.12±0.14^{f}	9.19±0.06^{e}
14	SS-128	13.14±0.15^{b}	13.77±0.14^{a}	13.25±0.21^{c}	14.27±0.05^{b}
	ΔLuxS/SS-128	12.08±0.19^{d}	13.40±0.05^{b}	12.96±0.01^{d}	13.08±0.14^{d}
24	SS-128	13.95±0.03^{a}	13.68±0.12^{a}	16.41±0.03^{a}	14.74±0.06^{a}
	ΔLuxS/SS-128	12.63±0.04^{c}	13.56±0.14^{a}	15.18±0.08^{b}	13.86±0.01^{c}

Means (n=3) in the same column without a common superscript letter (a-f) are statistically different, $P < 0.05$.

Table S2. Comparison of statistical results and reference genome.

Sample	SS128-1	SS128-2	SS128-3	*ΔluxS*/SS128-1	*ΔluxS*/SS128-2	*ΔluxS*/SS128-3
Total_reads	6648096	8189065	8086430	8077747	7498249	7251646
Mapped_reads	6253360	7708836	7613056	7620400	7078502	6851383
Map_PCG_Sn	74%	67%	69%	65%	64%	68%
Map_PCG_At	2%	2%	2%	3%	2%	2%
Map_rRNA_Sn	0%	0%	0%	0%	0%	0%
Map_rRNA_At	0%	0%	0%	0%	0%	0%
Map_tRNA_Sn	0%	0%	0%	0%	0%	0%
Map_tRNA_At	0%	0%	0%	0%	0%	0%
Map_miRNA_Sn	17%	22%	21%	22%	24%	20%
Map_miRNA_At	0%	1%	0%	1%	1%	1%
Map_un_region	8%	8%	7%	9%	9%	9%

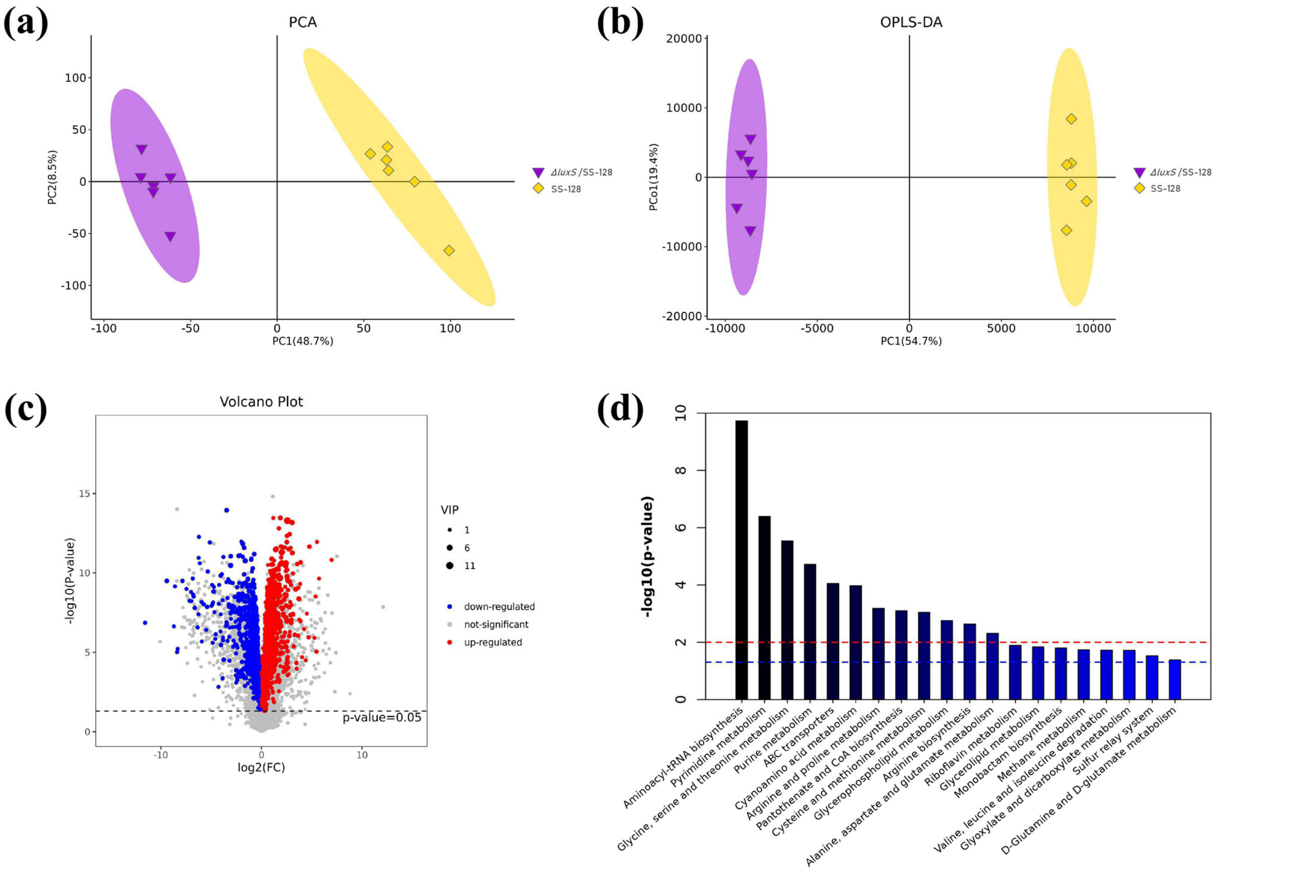

Figure S1. Changes in the metabolite contents compared the *luxS* mutant group with the control by LC-MS. The PCA (a), OPLS-DA (b), volcano plot (c) and metabolic pathway enrichment map-Top 20 (d) between *ΔluxS*/SS-128 and SS-128 under the cut off of *P*-value < 0.05 (*ΔluxS*/SS-128 vs SS-128). The red line and blue line in (d) indicate *P* = 0.01 and 0.05, respectively.
